# Does Perception of Dietary Fiber Mediate the Impact of Nutrition Knowledge on Eating Fiber-Rich Bread?

**DOI:** 10.3390/nu9111255

**Published:** 2017-11-16

**Authors:** Maria Królak, Marzena Jeżewska-Zychowicz, Marta Sajdakowska, Jerzy Gębski

**Affiliations:** Department of Organization and Consumption Economics, Faculty of Human Nutrition and Consumer Sciences, Warsaw University of Life Sciences (SGGW-WULS), 159C Nowoursynowska Street, 02-787 Warsaw, Poland; maria.krolak@gmail.com (M.K.); marta_sajdakowska@sggw.pl (M.S.); jerzy_gebski@sggw.pl (J.G.)

**Keywords:** dietary fiber, bread, nutrition knowledge, perception of dietary fiber, bread consumption

## Abstract

The average daily intake of fiber is still too low in relation to nutritional recommendations, as was found in several studies. Therefore, it is necessary to recommend ways to increase fiber intake in the diet. Increasing the consumption of bread rich in fiber as a substitute of white bread is one of the ways to increase fiber intake. The aim of this study was to find out whether nutrition knowledge and perception of dietary fiber affected the frequency of eating wholemeal bread and white bread fortified with fiber. The data were collected in 2014 through a cross-sectional quantitative survey that was performed under the Bioproduct project among a group of 1013 Polish adults. The associations between variables were investigated using multiple regression analysis. The respondents’ general knowledge on nutrition influenced their knowledge on fiber intake (correlation coefficient *r* = 0.30). Respondents with a greater knowledge perceived higher benefits of consuming cereal products that were fortified with fiber (*r* = 0.78), and attached greater importance to the information on the label (*r* = 0.39) as well. The nutrition knowledge determined the familiarity with fiber-enriched bread and the consumption of this product (*r* = 0.40) to a greater degree than the frequency of wholemeal bread consumption (*r* = −0.10). The respondents’ perception of dietary fiber was observed to play a partial mediation role between the knowledge on nutrition and the consumption of both kinds of breads, suggesting that it can be an important predictor of bread consumption. To increase the consumption of bread that is rich in fiber, emphasis should be laid on specific information on fiber, referring to food products as well as on individual’s perception of those products. The said information should be reinforced along with overall communication regarding nutrition to influence the bread-related decisions.

## 1. Introduction

Epidemiologic studies have shown that dietary fiber plays an important role in the prevention of obesity, type 2 diabetes, cancer, and cardiovascular disease (CVD) [1,2]. Regular consumption of dietary fiber, particularly that from cereal sources, may improve health through multiple mechanisms, such as reduction in lipid levels, weight regulation, improved glucose metabolism, blood pressure control, and reduction in chronic inflammation [3].

Nevertheless, the average daily intake of fiber in many population groups remains lower than fiber intake recommendations [4,5]. One of the causes of reduced dietary intake of fiber is the insufficient amount of wholemeal products in the diet [6,7] resulting, inter alia, from changing eating patterns and an increasing number of substitutes of such products [8]. Another reason why dietary fiber is not consumed in sufficient amounts may be the fact that high-fiber food is considered unpalatable [9]. The other factors that are perceived to have an adverse effect on the intake of fiber include, a higher price of wholemeal products in comparison with their refined equivalents, low availability of cereal products that constitute a good source of fiber, and the fact that these products do not meet sensory expectations of consumers [10].

According to some researchers, low intake of whole grains appears to be mainly because of insufficient knowledge about the beneficial effects of their consumption on health [6,10,11]. Contrarily, these deductions have not been confirmed by the results of other studies, indicating consumers’ adequate knowledge about dietary fiber [12]. However, some studies suggest that wholemeal products are not associated with health as much as fruits and vegetables, nor are they indicated as good sources of fiber [13,14]. Still, other researchers indicate that the majority of consumers are able to link fiber intake to its effects on the body and its preventive functions [14]. Consumers are aware of the beneficial influence dietary fiber and wholemeal products have on their health [15]; they also recognize products that are rich in fiber, and those with whole grains among them [13,14].

Because the awareness of the importance of a healthy lifestyle is increasing, breads containing whole grain, multi-grain, or functional components, such as fiber, are attracting a lot attention in the bakery industry [16]. But, in contrast to the positive effects on health, fiber-supplemented bread is still inferior in quality parameters when compared with those of white bread [17].

Earlier studies have attempted to identify the relations among demographic characteristics, food choice motives, and eating behaviors [18,19]. However, only some of them investigated the associations among those predicting variables [20]. In addition, no published research explored the mediating role of perception of dietary fiber intake between the nutrition knowledge and the behaviors of consumers.

The objective of this study was to investigate the associations among the knowledge on nutrition, the perception of dietary fiber, and the frequency of consuming wholemeal bread, as well as white bread fortified with fiber. Moreover, it was established whether the perception of dietary fiber played a mediating role between the knowledge on nutrition and behaviors that are related to the consumption of bread. 

## 2. Materials and Methods 

### 2.1. Study Sample

Data were collected in March 2014 through a cross-sectional quantitative survey under the Bioproduct project. According to the study design, recruitment and data collection were conducted by a research agency—BIOSTAT Group. Selection criteria of the sample considered the representativeness of the Polish population due to the province, the quota character by gender, education, and place of residence. Qualified to the interview were those above the age of 21 years and individuals who met other recruitment criteria, that is, consumption of at least two slices of bread a day and full or partial responsibility for the family’s grocery shopping. The computer-assisted personal interviewing (CAPI) technique was used to collect all data.

A pretest of the questionnaire through a pilot study (*n* = 50) was performed within the population of interest. This study was conducted according to the guidelines laid down in the Declaration of Helsinki. The Bioethics Committee of the Faculty of Medical Sciences, University of Warmia and Mazury in Olsztyn, approved the protocol of the study on the 17 June 2010, Resolution No. 20/2010. Written informed consent was obtained from all of the participants.

### 2.2. Outcome Variables

To evaluate behaviors involving the use of bread rich in dietary fiber, questions concerning (1) the frequency of eating wholemeal bread; and (2) the familiarity with white bread fortified with fiber and the consumption of this product were asked. The frequency of eating wholemeal bread was measured on a 6-point scale, where 1 stands for once a month or less, 2—once a fortnight, 3—once a week, 4—2–3 times a week, 5—4–5 times a week, 6—once a day or more. The familiarity with white bread fortified with fiber and the consumption of this product were measured on a 4-point scale, described as “I do not know such a product and I do not want to try it” (1), “I do not know such a product, but I would like to try it” (2), “I know this product, but I do not wish to buy it, nor consume it” (3), “I know this product and I consume it” (4)*.*

### 2.3. Explanatory Variables

The explanatory variables included the knowledge on nutrition; the perception of dietary fiber including the knowledge on fiber, the perception of benefits of eating cereals fortified with fiber, and the perception of the importance of bread labeling concerning the fiber content, as well as demographics, such as gender, age, and education.

Nutritional Knowledge Scale (NKS), developed by the Commission of Behavioral Determinants of Nutrition from the Polish Academy of Sciences [21] was used to assess the participants’ nutrition knowledge. The respondents were asked to give opinions on each of the 26 statements from the NKS, choosing one of the three following options: 1—I do not agree, 2—I agree, and 3—I do not have an opinion. The data was recoded based on the agreement or disagreement with the statement for correctness of the respondent’s answer (1—correct answer, 2—incorrect answer). When the nutritional knowledge of the respondents was assessed, re-recoding was necessary. One point was awarded for a correct answer (1), whereas no points were awarded for an incorrect answer (2), or for the inability to decide the correctness of the statement (3). The sum of scores of all the 26 items of NKS was calculated for each of the participants. The higher the score was, the greater the respondent’s knowledge on nutrition was assumed to be.

Consumers’ knowledge on dietary fiber was measured with five statements, each with a 5-point scale, described starting from (1) strongly agree to (5) strongly disagree (Table 1). The sum of the scores of the five items was calculated for each participant. Moreover, the opinions about the benefits of eating cereals fortified with fiber were measured with three statements on a 5-point scale, each described starting from (1) strongly agree to (5) strongly disagree. The opinion about the importance of fiber labeling was measured on a 5-point scale described starting from (1) completely unimportant to (5) highly important.

### 2.4. Statistical Analysis

Statistical analysis was conducted using IBM SPSS Statistics for Windows, version 21.0 (IBM Corp, Armonk, NY, USA). Descriptive statistics, including frequency distributions and cross-tabulations, were performed. Cronbach’s alphas and inter-item correlations were calculated to determine the internal consistency of scales regarding the assessment of knowledge on nutrition, knowledge on dietary fiber, and benefits of eating cereals that were fortified with fiber. Relations among different parameters were assessed by calculating Pearson’s correlation coefficients. A *p*-value of < 0.05 was determined as significant.

A multiple regression analysis was applied to verify the associations among the variables, and the mediating role of perception of dietary fiber between nutrition knowledge and behaviors that are related to the consumption of bread. In the first three models, demographics and knowledge on nutrition were treated as independent variables and were regressed on the variables knowledge on dietary fiber (Model 1), benefits from eating cereals fortified with fiber (Model 2), and the importance of fiber content labeling (Model 3). The results of these three models tested the correlations between the knowledge on nutrition and the perception of dietary fiber (three variables treated independently). In the next two models, all of these factors (demographics, knowledge on nutrition, knowledge on dietary fiber, benefits from eating cereals fortified with fiber, and importance of fiber labeling) were treated as independent variables and regressed on the variables familiarity with white bread fortified with fiber and eating behaviors regarding this product (Model 4) and the frequency of eating wholemeal bread (Model 5). These models tested the correlations of the independent variables with the eating behaviors regarding both the breads.

## 3. Results

More than half of the sample population were women (53.4%). The average years was 47.6 years (standard deviation 4.43). About 35.5% of the respondents were characterized by education lower than secondary, 36.5% had secondary education, and 27.9% graduated from university. About 38.4% of the respondents lived in rural areas, whereas 61.6% of participants lived in urban areas. 

The sum of the scores representing the nutrition knowledge of the respondents ranged from 0 to 22 points. Extreme results were achieved by 25 people—22 respondents obtained 0 points and 3 respondents obtained 22 points. The mean value of nutrition knowledge among the respondents was 11.01, the standard deviation being 4.43. The alpha coefficient for NKS was 0.79.

The respondents’ opinions on dietary fiber are presented in Table 1.

The alpha coefficients for consumers’ knowledge on dietary fiber scale (0.84), and for benefits of eating cereals fortified with fiber scale (0.83) have shown good internal reliability of the scales. 

The percentage distribution of respondents with varying answers regarding familiarity with white bread fortified with fiber and the consumption of the same was as follows: “I do not know such a product and I do not want to try it”—6.8%; “I do not know such a product, but I would like to try it”—32.9%; “I know this product, but I do not wish to buy it, nor consume it”—20.4%; and, “I know this product and I consume it”—39.9%. The frequency of consuming wholemeal bread was once a month or less—12.3%, once a fortnight—18.5%, once a week—20.9%, 2–3 times a week—21.6% 4–5 times a week—18.5%, and once a day or more often—8.2%.

Table 2 presents the correlations between the knowledge on nutrition, the perception of dietary fiber treated as three variables, and behaviors involving the consumption of fiber-rich bread. 

Table 3 summarizes the results of multiple regression analysis. 

The sociodemographic characters, such as gender (dummy coded), age, place of residence (dummy coded), level of education (dummy coded), and nutrition knowledge were entered into Models 1–3. In each of them, nutrition knowledge was significantly associated with all of the variables describing the perception of dietary fiber, but the correlations were found to be weak. Benefits from eating cereals fortified with fiber had the best predictability from nutrition knowledge. Importance of fiber content labeling on a product had the least predictability from nutrition knowledge.

In Models 4 and 5 independent variables were entered into the models in two steps. In step 1, the variables gender (dummy coded), age, place of residence (dummy coded), level of education (dummy coded), and nutritional knowledge were entered. In step 2, the variables knowledge on fiber, benefits from eating cereals fortified with fiber and the importance of fiber content labeling were entered into both the models. For white bread fortified with fiber in step 1 of Model 4, the sociodemographic variables and nutrition knowledge accounted for 18% of the variance in familiarity with white bread fortified with fiber and the consumption of this product. In step 2, after adding the three factors of perception of dietary fiber, a 13% increase in the *R*^2^ was observed. Thus, the prediction was a total of 31% of the variance in familiarity with and consumption behaviors regarding white bread fortified with fiber. Secondary and higher education, nutrition knowledge, knowledge on fiber, and importance of fiber content labeling could largely affect familiarity with white bread fortified with fiber and consumption behaviors regarding this product. It indicates a partial mediation of knowledge on dietary fiber and the importance of fiber content labeling between nutrition knowledge and familiarity with white bread enriched with fiber, as well as the consumption of this product.

In the case of the frequency of eating wholemeal bread (Model 5), the sociodemographic variables and nutritional knowledge accounted for only 2% of the variance. In the step 2, after adding three variables describing the perception of dietary fiber, only a 4% increase in *R*^2^ was observed. Gender, knowledge on fiber, and benefits from eating cereals fortified with fiber could significantly predict eating behaviors. Nevertheless, the model cannot be used to predict the frequency of the consumption of wholemeal bread because of a very low level of the explained variance.

## 4. Discussion

Epidemiological and clinical studies show that dietary fiber consumption results in a reduced risk of obesity, type 2 diabetes, cancer, and CVD [2,22]. Nevertheless, the possible health benefits deriving from fiber consumption depend on the level of fiber intake. WHO recommended a fiber intake of 25 g and higher. Recommendations of total fiber intake for an average population are differentiated in some countries and range from 20 g to 45 g [5]. The average daily intake of fiber is too low in relation to nutritional recommendations among adults in Europe, Australia, New Zealand, and the USA [5], which significantly reduces the potential health-promoting effects of dietary fiber.

The amount and composition of fiber differ among different types of foods and the intake of fiber from such products is varied [5]. Cereals are one of the major sources of dietary fiber, contributing to about 50% of the fiber intake in Western countries. Vegetables provide 30–40% of dietary fiber, about 16% comes from fruits, and the remaining 3% from other sources [23,24].^.^ Górecka et al. [25] showed that in Poland in 2000–2009, some of the main sources of dietary fiber were cereal products (41.5%). Vegetables ranked second, with 26.4%. 

Knowledge about nutrition and health is seen as an important determinant of food choices [20,26,27]. The more consumers know about food and nutrition, the more likely they are to exhibit nutritional behaviors that are beneficial for their health. The positive relation between nutrition knowledge and pro-health behaviors has been observed in many studies [28,29], although in some studies it has not been confirmed [30]. Its absence can be a result of factors of a greater importance other than nutrition knowledge, especially ones related to the external environment, which is confirmed in the studies about food selection factors [28,31,32].

In our research, significant correlations between nutrition knowledge, consumption of wholemeal bread and bread fortified with fiber, and the perception of dietary fiber were indicated. In the case of the frequency of eating wholemeal bread, these correlations were found to be negative. The greater the knowledge on nutrition and the better the perception of dietary fiber, the less frequent was the consumption of wholemeal bread reported by the respondents. The result is contrary in comparison with the results of other studies. Most of the previous studies have indicated that increasing nutritional awareness boosted consumers’ interest in healthier food and supported healthier nutritional behaviors [33], which include eating wholemeal bread. Nevertheless, higher nutrition knowledge is not necessarily related to having satisfactory knowledge on specific nutrition issues. The NKS used to assess the nutrition knowledge included only one statement that was related to whole grains usage. Some qualities of wholemeal bread confirmed in other studies, as the lower scores in regard to sensory attributes of wholemeal bread when compared with white bread [9], the higher price of wholemeal bread [22], as well as its low availability [11,26] may constitute reasons of the negative correlation. The findings from Eurobarometer concerning food-related risks indicated that the European consumers underestimate the significance of the effects their eating habits have on their health [33], which may explain the results obtained in the study concerning the consumption of wholemeal bread. In Poland, both a much higher availability of white bread and a significantly higher price of wholemeal bread can affect the less-frequent consumption of the wholemeal bread. The increase in nutrition knowledge does not compensate for economic constraints, although studies on this issue have not been conducted yet. 

Knowledge on nutrition can be treated as a direct determinant of nutritional behaviors [34], but it may also be viewed as a factor influencing other choice motives, both cognitive and affective ones. Our study covered not only the nutritional knowledge, but also other cognitive motives for choosing bread, including knowledge about dietary fiber, perceived benefits from eating cereals enriched with fiber, and perceived importance of fiber content labeling [26,32,35,36]. The greater the nutrition knowledge the respondents had, the more they perceived the benefits of consuming cereals with added fiber. They also had a greater knowledge about dietary fiber. Similar results were obtained in other studies [9]. Although to the smallest extent, but in a statistically significant way, the nutritional knowledge correlated with the declared importance of fiber labeling.

The mediating role of the perception of dietary fiber resulted in the lack of an inverse relation between nutrition knowledge and the frequency of consumption of wholemeal bread. It was therefore concluded that the knowledge on dietary fiber and the perceived benefits of consuming cereal with added fiber constituted more important predictors of the frequency of consuming wholemeal bread than nutrition knowledge alone. A greater knowledge about dietary fiber translated into more frequent consumption of wholemeal bread, which was also confirmed in other studies [28]. Perceived benefits from eating white bread with added dietary fiber were associated with a lower frequency of eating wholemeal bread. People who consumed wholemeal bread perceived less benefits from eating white bread that is enriched with dietary fiber, which can be explained by the greater importance of naturalness for the consumers of wholemeal products [22].

Earlier studies have shown that consumers are aware of the health benefits from eating wholemeal foods and therefore they may not perceive the consumption of an alternative functional product, such as dietary fiber, added to white bread as an effective way to avoid disease [28], which confirms an inverse weak relation between the consumption of both types of breads. Helleyer et al. [35] observed that participants preferred wholegrain bread as a food that provides health benefits, while simultaneously being a source of functional ingredients, such as fiber. Consumers were also willing to pay more for a wholegrain bread than other types of bread, including white bread containing functional ingredients. Thus, despite the skills of the bakery industry related to the production of a healthier white bread, consumers did not appear to be willing to pay more for this product when compared with bread made up of wholegrain. However, in our research, the respondents declared a relatively high interest in white bread enriched in fiber. These results need to be confirmed in further studies, including the exploration of sensory evaluation of bread with added fiber.

Many studies have shown that demographic variables, such as gender, age, and education influence individuals’ food choice motives and nutritional behaviors [18,37,38,39]. The nutrition knowledge and attitudes toward food differ among different sociodemographic groups [40], which has been confirmed in the present study. Research by Ginon et al. [41] indicated that the older consumers who careful about their intake of fiber in the diet were more likely to accept a product labeled as containing fiber and showed a greater willingness to purchase it, even if it meant paying a premium price. In our study, age as well as education, did not significantly differ with the frequency of consumption of wholemeal bread, and neither did the opinions about the importance of fiber content labeling. However, familiarity with bread with added fiber and its consumption decreased with age, whereas there was an increase in education combined with a greater familiarity with such a bread and a higher consumption of this product. The older the respondents were, the lesser was their nutrition knowledge, including the knowledge about dietary fiber. Moreover, they perceived less benefits from consuming cereal products that are enriched in dietary fiber. The increase in the level of education was connected with higher nutrition knowledge, greater knowledge about dietary fiber, and perception of more benefits of consuming cereal products that are fortified with fiber.

Men consumed wholemeal bread less frequently than women; they were also less familiar with white bread that is enriched in fiber. Moreover, nutrition knowledge and knowledge about dietary fiber was lower among men in comparison with that among women. They also perceived less benefits of consuming cereal with added fiber and attributed less importance to fiber content labeling. The differences between men and women regarding their knowledge about nutrition, as well as nutrition behaviors, were reported in several studies [32,33,42], which is mainly explained by a greater involvement of women in providing food for the family [43] and a greater importance attached by women to their own appearance [44].

Paying attention to fiber content labeling is important while choosing food, especially referring to food with a modified composition. This information about food content is of crucial importance for consumer food choice behavior and may affect the food choice that is favorable to health [32,45,46]. Nevertheless, findings from other studies investigating baguette consumption after fiber content was made available, found no significant effects on the willingness of consumers to purchase the baguettes [41]. Similar results were reported by Mialon et al. [15] for white bread that is enriched in fiber. However, according to other studies, fiber content labeling fosters a positive attitude toward the product and provides an increase in its perceived value [41,47]. In our study, a positive correlation between the perceived importance of fiber content labeling and the consumption of white bread enriched in fiber has been demonstrated.

This study has some limitations. One of them concerns the sociodemographics characteristics that have not been taken into account, while the sample was being selected. It is a limitation that no health measures were included, such as CVD or dyslipidemia. Moreover, the sample included only those solely or jointly responsible for the family’s grocery shopping. In addition, eating behaviors for both of the products were measured in different ways. Low availability of white bread fortified with fiber did not allow for using the same frequency categories implemented for wholemeal bread. Further studies involving the use of another measure of nutrition knowledge may confirm the obtained results, indicating an inverse correlation between nutrition knowledge and the frequency of eating wholemeal bread. Finally, only cognitive motives concerning dietary fiber were included as mediators of bread choice. There is a need to include emotional and sensory factors that may also modify the links between nutrition knowledge and the frequency of bread consumption.

## 5. Conclusions

Those with a higher nutritional knowledge were more likely to know about the nutritional benefit of eating a diet rich in fiber. Moreover, they perceived more benefits of consuming cereal products fortified with fiber and attached a higher importance to fiber content labeling. Familiarity with bread enriched in fiber and its consumption was highly dependent on higher nutrition knowledge in comparison with the frequency of consuming wholemeal bread. The nutrition knowledge and the perception of dietary fiber determined the familiarity with white bread enriched in fiber with and the consumption of this product to a greater degree than the frequency of consumption of wholemeal bread. These motives played a partial mediation role between the nutrition knowledge and the consumption of both kinds of breads.

The findings confirmed that the relation between eating behaviors and nutrition knowledge is also complex for wholemeal bread. Impact of consumers’ nutrition knowledge on eating behaviors is mediated by the perception of particular food ingredients. These results are important for targeting public nutrition and public health education programs, as well as for marketing communication. They underline the need for an individual approach considering both the food itself and the psychosocial characteristics of the consumers when the aim is to bring about change in their behaviors. The results may provide important data for those who develop educational strategies and interventions. To change current behaviors, nutrition education needs to include, besides overall nutrition information, knowledge on specific food components and on individual’s perception of these components. The differences in factors determining the behaviors that are related to wholemeal bread and white bread enriched in fiber require communication adapted to the specificity of the product.

## Figures and Tables

**Table 1:** Respondents’ opinions on dietary fiber.

Statements	Scale *	Mean	SD **	Alpha
*Knowledge on dietary fiber*				
Fiber helps maintain proper blood cholesterol level	1–5	3.49	1.07	0.83
Fiber satisfies the sensation of hunger	1–5	3.52	1.02	
Fiber accelerates the movement of digesta through intestines	1–5	3.78	0.99	
Wholemeal bread is a good source of fiber	1–5	3.82	0.99	
One should control the amount of fiber consumed	1–5	3.30	1.07	
*Benefits of eating cereals fortified with fiber*				
Cereals fortified with fiber facilitate leading a healthy lifestyle	1–5	3.70	0.94	0.85
Cereals can diminish negative effects of an unhealthy diet	1–5	3.61	0.95	
I can prevent illnesses by eating such products on a regular basis	1–5	3.52	0.95	
*Importance of fiber labeling*				
Information about the fiber content on the label of bread is important to me	1–5	3.65	1.11	-

* Knowledge on fiber and benefits from eating are measured on a 5-point scale ranging from 1—strongly disagree to 5—strongly agree. Importance of fiber labeling is measured on a 5-point scale ranging from 1—completely unimportant to 5—highly important. ** Standard deviation.

**Table 2:** Means, standard deviations, and correlations between variables (correlation coefficients).

Items	No. of Items	Mean	SD.	1	2	3	4	5	6	7	8	9
Gender ^a^	1	0.53	0.49									
Age ^b^	2	47.62	16.47	−0.02								
Education ^c^	3	3.05	1.17	−0.03	−0.50 **							
Knowledge on nutrition ^d^	4	11.01	4.35	−0.07 *	−0.09 **	0.28 **						
Knowledge on fibre ^e^	5	3.65	0.82	−0.11 **	−0.11 **	0.17 **	0.30 **					
Benefits from eating cereals fortified with fiber ^f^	6	3.61	0.81	−0.08 **	−0.12 **	0.19 **	0.33 *	0.78 **				
Importance of fiber labeling ^g^	7	3.65	1.11	−0.10 **	−0.03	0.05	0.11 **	0.39 **	0.39 **			
Frequency of eating whole meal bread ^h^	8	3.59	1.50	−0.07 *	−0.02	−0.01	−0.10 **	−0.18 **	−0.15 **	0.05		
Familiarity with and consumption of white bread fortified with fiber ^i^	9	2.93	0.99	−0.09 **	−0.10 **	0.26 **	0.40 **	0.45 **	0.40 **	0.29 **	−0.06 *	

^a^ gender: male—0, female—1; ^b^ age—in years; ^c^ education: lower than secondary—1; secondary—2; university—3; ^d^ knowledge on nutrition was measured as a sum of correct answers; ^e^ knowledge on fiber was measured on a 5-point Likert-type scale ranging from 1—strongly disagree to 5—strongly agree; ^f^ benefits from eating were measured on a 5-point Likert-type scale ranging from 1—strongly disagree to 5—strongly agree; ^g^ importance of fiber labeling: measured on a 5-point scale ranging from 1—completely unimportant to 5—highly important; ^h^ frequency of eating whole meal bread: measured on a 6-point scale, where 1—once a month or less, 2—once a fortnight, 3—once a week, 4—2–3 times a week, 5—4–5 times a week, 6—once a day or more; ^i^ familiarity with white bread fortified with fiber and eating this product: measured on a 4-point scale, where 1—I do not know such product and I do not want to try it, 2—I do not know such product, but I would like to try it, 3—I know this product, but I do not wish to buy it nor consume it, 4—I know this product and I consume it. * *p* < 0.05; ** < 0.01; SD—standard deviation.

**Table 3:** Summary of results from multiple regression analysis testing the mediation of perception of dietary fiber when predicting eating frequency of white bread fortified with fiber and wholemeal bread.

	Perception of Dietary Fiber ^f^			Familiarity with and Eating Behaviors of White Bread Fortified with Fiber ^i^ (Model 4)		Frequency of Eating Wholemeal Bread ^j^ (Model 5)	
	Knowledge on Fiber ^f^ (Model 1)	Benefits from Eating ^g^ (Model 2)	Importance of Fiber Labelling ^h^ (Model 3)				
				Step 1	Step 2	Step 1	Step 2
*Demographics*							
Gender ^a^	0.09 **	0.06	0.09 **	0.06 *	0.02	0.08 *	0.08 **
Age ^b^	−0.06	−0.05	−0.02	0.01	0.03	−0.04	−0.05
Secondary education ^c^	0.04	0.03	−0.02	0.15 ***	0.14 ***	−0.05	−0.04
Higher education ^d^	0.06	0.10 *	0.01	0.16 ***	0.14 ***	0.01	0.02
*Knowledge on nutrition* ^e^	0.27 ***	0.29 ***	0.10 **	0.35 ***	0.26 ***	−0.12 ***	−0.06
Knowledge on fiber ^f^					0.28 ***		0.13 ***
Benefits from eating ^g^					0.02		−0.18 ***
Importance of fiber labelling ^h^					0.14 ***		−0.06
*R*^2^	0.11	0.12	0.02	0.18	0.31	0.02	0.06
F ^i^	23.59 ***	28.20 ***	4.58 ***	44.54 ***	55.77 ***	4.17 ***	8.31 ***

**^a^** gender: male—0, female—1; ^b^ age: in years; ^c^ secondary education: not secondary education—0, secondary education—1; ^d^ higher education: not higher education—0, higher education—1; ^e^ knowledge on nutrition: as a sum of correct answers; ^f^ knowledge on dietary fiber was measured on a 5-point scale ranging from 1—strongly disagree to 5—strongly agree; ^g^ benefits from eating were measured on a 5-point scale ranging from 1—strongly disagree to 5—strongly agree; ^h^ importance of fiber labelling: measured on a 5-point scale ranging from 1—strongly unimportant to 5—strongly important; ^i^ familiarity and eating white bread fortified with fiber: measured on a 4-point scale, where 1—I do not know such product and I do not want to try it, 2—I do not know such product, but I would like to try it, 3—I know this product, but I do not wish to buy it nor consume it, 4—I know this product and I eat it; ^j^ frequency of eating whole meal bread: measured on a 6-point scale, where 1—once a month or less, 2—once a fortnight, 3—once a week, 4—2–3 times a week, 5—4–5 times a week, 6—once a day or more; ^i^ F—value from test of overall significance; * *p* < 0.05; ** < 0.01; *** *p* < 0.001.
